# A Resource to Infer Molecular Paths Linking Cancer Mutations to Perturbation of Cell Metabolism

**DOI:** 10.3389/fmolb.2022.893256

**Published:** 2022-05-18

**Authors:** Marta Iannuccelli, Prisca Lo Surdo, Luana Licata, Luisa Castagnoli, Gianni Cesareni, Livia Perfetto

**Affiliations:** ^1^ Department of Biology, University of Rome Tor Vergata, Rome, Italy; ^2^ Fondazione Human Technopole, Milan, Italy

**Keywords:** metabolic pathway, rate limiting enzyme, cancer, causal interaction, network, SIGNOR

## Abstract

Some inherited or somatically-acquired gene variants are observed significantly more frequently in the genome of cancer cells. Although many of these cannot be confidently classified as driver mutations, they may contribute to shaping a cell environment that favours cancer onset and development. Understanding how these gene variants causally affect cancer phenotypes may help developing strategies for reverting the disease phenotype. Here we focus on variants of genes whose products have the potential to modulate metabolism to support uncontrolled cell growth. Over recent months our team of expert curators has undertaken an effort to annotate in the database SIGNOR 1) metabolic pathways that are deregulated in cancer and 2) interactions connecting oncogenes and tumour suppressors to metabolic enzymes. In addition, we refined a recently developed graph analysis tool that permits users to infer causal paths leading from any human gene to modulation of metabolic pathways. The tool grounds on a human signed and directed network that connects ∼8400 biological entities such as proteins and protein complexes via causal relationships. The network, which is based on more than 30,000 published causal links, can be downloaded from the SIGNOR website. In addition, as SIGNOR stores information on drugs or other chemicals targeting the activity of many of the genes in the network, the identification of likely functional paths offers a rational framework for exploring new therapeutic strategies that revert the disease phenotype.

## Introduction

The cell responses to environmental stimuli or genetic perturbations are modulated by the signalling network and by the cell metabolic landscape. In growing cells, the metabolite composition of the cell milieu not only supports anabolic processes but also affects signal transduction and, as a consequence, cell fate and function. Signalling and metabolism crosstalk as activation of canonical cellular pathways often regulate metabolic-enzyme concentration or activity (Ward and Thompson 2012). On the other hand, small molecules that are the product of metabolism may affect the activity of signalling proteins and some metabolic enzymes fulfil distinct signalling functions (Pietrocola et al., 2015; Frezza 2017; Godfrey and Kornberg 2020). Thus, it is becoming increasingly apparent that signal transduction and metabolism are not two separate processes but are parts of a single large network of causal interactions.

These considerations are particularly relevant in cancer biology where alterations in oncogenes, onco-suppressors (TSG) or additional modifier genes play important roles in redirecting cell metabolism to fulfil the biosynthetic demands associated with proliferation (Warburg 1956; Pavlova and Thompson 2016; Farhadi et al., 2020). In addition, tumour metabolism can affect resistance to chemo-, radio-and immunotherapies as well as targeted therapies (Zaal and Berkers 2018).

A complete and holistic understanding of the mechanisms that lead to cell transformation is essential to devise pharmacological regimens that revert the disease phenotype by targeting metabolic vulnerabilities (Luengo et al., 2017; Faubert et al., 2020).

A wide range of computational strategies have been proposed to characterise the capabilities of biological systems through analysis of metabolic network models (Antoniewicz 2021). These include flux balance analysis (FBA), metabolic flux analysis (MFA) and 13C-metabolic flux analysis (13C-MFA), which exploit prior metabolic networks and make them context specific by retaining only the reactions that fit with experimental results. More recently, the group of Professor Saez-Rodriguez has released COSMOS (Causal Oriented Search of Multi-Omic Space) (Dugourd et al., 2021). COSMOS is a complex computational method that makes use of prior knowledge of signaling and metabolic networks to integrate multi-omic datasets (e.g., phosphoproteomics, transcriptomics, and metabolomics). The ultimate goal of COSMOS is the one of generating novel hypotheses relevant for the experimental setup.

**FIGURE 1:**
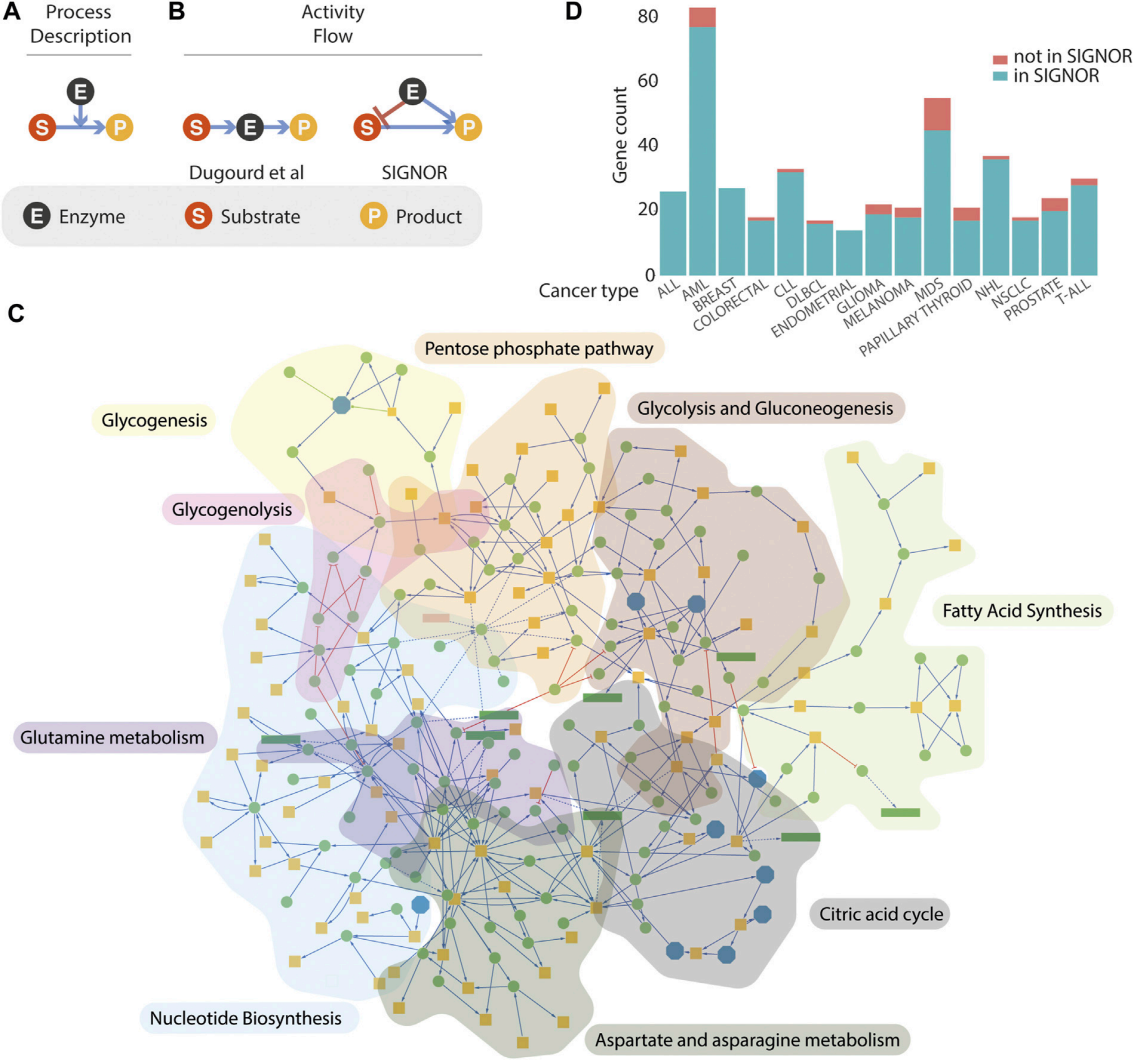
Representation of metabolism in SIGNOR. **(A,B)** three models that can be used to represent enzymatic reactions. See text for details. **(C)** Graph representations of the metabolic pathways annotated in the SIGNOR databases. Orange square represents small organic molecules that participate in the enzymatic interactions, Green circles are enzymes that catalyse the reactions or other proteins modulating their activities. Reactions are arbitrarily organized into nine metabolic pathways that are identified by a different background colour. **(D)** Coverage in the SIGNOR database of the cancer driver genes annotated in the Cancer Gene Census. The height of each green bar is proportional to the number of cancer genes in the SIGNOR human network that are annotated to 15 specific tumour types. The fraction of cancer genes for which we couldn’t find causal information is in orange. T-ALL, T cell acute lymphoblastic leukaemia; NSCLC, Non-small-cell lung cancer; NHL, non-Hodgkin lymphoma; MDS, myelodysplastic syndrome; DLBCL, diffuse large B-cell lymphoma; CLL, chronic lymphocytic leukaemia; AML, acute myeloid leukaemia; ALL, acute lymphocytic leukaemia.

All the methods described leverage on prior knowledge models extracted from the literature or from existing repositories. Over recent years several resources have endeavoured to represent published experimental information on signalling or metabolism in a computer readable format (e.g. Recon3D, Reactome, KEGG (Jassal et al., 2020; Kanehisa et al., 2017; Brunk et al., 2018, 3). The complexity of biological interactions is captured by the different resources by two main types of simplified models, often referred to as “process descriptions” (PD) and “activity-flow” (AF) (Figure 1) (Le Novère and Nicolas, 2015; Touré et al., 2020). The two model types differ in structure and support different types of analysis (Cesareni, Sacco, and Perfetto 2021). Recon3D and Reactome integrate signalling and metabolism adopting exclusively the PD model (Figure 1A), whereas KEGG adopts the AF and the PD models to depict signalling and metabolic pathways respectively. The absence of a unique representation has frustrated attempts to compare and integrate the different datasets and the integration of signalling and metabolism is hampered by the lack of a unique graph model that satisfactorily represents the crosstalk of signalling and metabolism. Dugourd and colleagues, in the effort to develop COSMOS and to embed metabolic activities into activity flow graphs, have faced the challenge to map PD interactions annotated in Recon3D onto an AF representation (Dugourd et al., 2021).

Here we aim at proposing a new representation of metabolic reactions that favours their integration with activity-flow signalling models. In addition, we introduce a novel tool allowing inference of the impact of any gene variant on a selection of key metabolic pathways. To this end, we embarked on a curation effort aimed at capturing in the SIGNOR database (Licata et al., 2020) 1) metabolic pathways that are deregulated in cancer and 2) interactions connecting oncogenes and tumour suppressors to metabolic enzymes. This dataset can be freely accessed via the CancerGeneNet resource (https://signor.uniroma2.it/CancerGeneNet) (Iannuccelli et al., 2020).

## Results

### Integration of Metabolism Into a Causal Model

Activity-flow (AF) models offer a simple, albeit powerful, framework to represent the relationships governing the crosstalk between biological entities (Touré et al., 2020; Cesareni et al., 2021). They are particularly suited to support Boolean modelling and to simulate the propagation of signals in pathways where the activity of an entity is modulated either positively or negatively by the activity of upstream entities (Kauffman 1969). Metabolic reactions on the other hand are better represented by process-description models (Figure 1A) where the enzymatic reaction is pictured as a directed edge symbolizing the chemical transition and linking the substrates to the products. The enzyme activity is represented as a second edge positively impacting on the chemical transition from substrates to products. The latter are employed in quantitative modelling approaches that are based on ordinary differential equations. Recently, Dugourd and colleagues have proposed a different graph representation of metabolic reactions (Figure 1B) where the substrate is linked by an “activating” edge to the enzyme that in turn connects to the substrate (Dugourd et al., 2021). This model permits the integration of metabolic reactions into a causal signalling network. However, it ignores information about the specific link between each substrate and the corresponding products of the enzymatic reaction. To address this point, we present an alternative model to integrate enzymatic reactions into logic models (Figure 1B). According to this representation, both the substrate and the enzyme catalysing the reaction connect to the product with activating edges, as they are both necessary to yield the product. In addition, a negative edge linking the enzyme to the substrate signals that the activity of the enzyme leads to substrate consumption.

### Curation of Metabolic Pathways in SIGNOR

SIGNOR was originally conceived as a resource to link signalling proteins via causal relationships (Perfetto et al., 2016). It later extended its scope to include additional biological entities (protein families, complexes, siRNA, small molecules, etc.) that could be relevant to represent signal propagation and its impact on phenotypes. Initially, metabolism has not been a focus of the project as enzymatic reactions could poorly be represented by the model adopted in the resource. The development of the project, as described in this report, was meant to overcome this limitation. Over recent months, more than 1300 metabolic reactions and 120 metabolic enzymes have been integrated into the human causal interactome. The enzymatic reactions have been organized into nine pathways, concentrating on pathways whose activities are deregulated in cancer cells (glycolysis and gluconeogenesis, citric acid cycle, glycogenesis, glycogenolysis, aspartate and asparagine metabolism, fatty acid synthesis, glutamine metabolism, nucleotide biosynthesis, pentose phosphate (Joly, Chew, and Graham 2021). In addition, we have identified, in each pathway, key enzymes whose activities are rate limiting and are therefore more likely to affect the metabolic flux when their concentrations or activities are modulated (Supplementary Table S1). The nine pathways merge into a single completely connected large metabolic network embedded into the human causal network (Figure 1C).

### Increased Coverage of Cancer Genes

Over the past couple of years cancer genes have been a target of the curation effort by the SIGNOR team. In 2019 a related resource CancerGeneNet (Iannuccelli et al., 2020) was developed to facilitate the inference of signalling paths connecting cancer genes to cancer hallmark phenotypes (Hanahan and Weinberg 2011). At that time 142 genes listed in the Cancer Gene Census (CGC).

(Sondka et al., 2018) could not be integrated into the human network as we couldn’t retrieve from the literature any experimental evidence of causal relationships with other entities in the network. More recently the publication of causal evidence for 16 additional genes in the cancer gene census allowed their incorporation into the SIGNOR human network. Presently over 84% of the 729 CGC genes are connected by causal relationships to the SIGNOR network. The bar diagram in Figure 1D reports the coverage in SIGNOR of the genes annotated to 15 tumour classes in the CGC.

Recently, two consortia from different institutes have reported CRISPR-Cas 9 screening that yielded lists of prioritized therapeutic-gene targets (Tsherniak et al., 2017; Behan et al., 2019). The results of the two independent screens are highly concordant indicating that this approach yields robust findings (Dempster et al., 2019). The gene hits of the screens define a genetic background that favours tumour growth. To increase the coverage of the causal network connecting genes that have an impact on cancer we have screened the literature looking for evidence of causal interactions between genes in these lists and other genes in the human network. This effort contributed to the integration in the human causal network of approximately 276 cancer dependency genes.

### Inferred Impact of Cancer Genes on Metabolism

Some cancer genes, when mutated, modify cell metabolism to facilitate the biosynthesis of the macromolecules and organelles required for assembling new cells. However, in many cases, the underlying molecular mechanisms are poorly understood.

The integration of metabolic reactions into the human signalling network permits to connect, via causal paths, the activity of cancer genes to metabolic pathways. To this end we have built, in the resource CancerGeneNet, a graph tool that searches for short weighted-signed-directed paths linking two nodes in the human causal network. The tool allows one to infer how likely it is that a query protein (or a list of proteins) modulates the activity or expression of key rate-limiting enzymes in nine metabolic pathways.

Briefly, the tool is based on a selection of approx. 13,000 paths that significantly link genes in SIGNOR to key rate-limiting metabolic enzymes. To obtain this dataset we filtered the entire SIGNOR interactome by applying the three following steps: 1) we identified paths connecting, with four causal steps or fewer, any gene in SIGNOR to metabolic pathways; 2) As each path is associated with a distance score (see Methods), for each considered metabolic pathway we could draw a distance distribution and calculate a Z score for each gene distance; 3) genes with a Z score < −1.96 (*p*-value <0.05) were considered to be likely to have an impact on the pathway.

Genes with a Z score < −1.96 (*p*-value <0.05) were considered to be likely to have an impact on the pathway. Next, we have asked whether the list of genes annotated as oncogenes or tumour suppressor genes by the CGC resource were enriched for genes that affect metabolism. The result of this analysis showed that 40% (97/245) of the genes annotated as oncogenes are significantly close to metabolic pathways. This is significantly (*p*-value = 1.9E-05) higher than observed in 1000 collections of randomly sampled genes (average 21%) and supports the notion that genes that are found preferentially mutated in tumours are more likely to affect metabolism than random genes.

**FIGURE 2:**
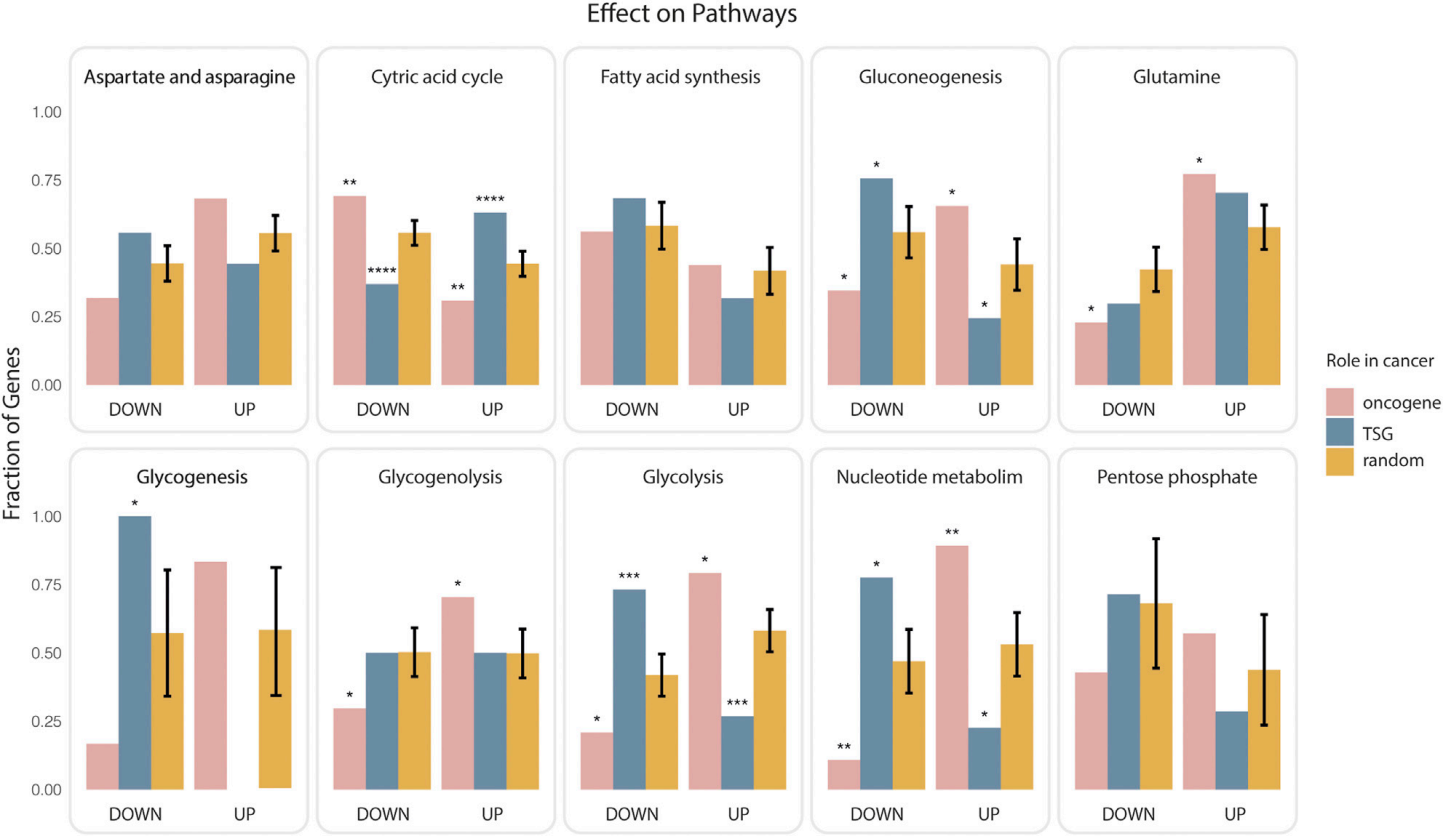
Impact of cancer genes on metabolism. Oncogenes or onco-suppressors (TSG) that were found to be significantly closer to ten metabolic pathways were classified as activators or inhibitors depending on whether the paths of causal interactions that permit joining the gene and the rate limiting enzymes contained an even or odd number of inhibitory steps. The fraction of activating (UP) or inhibiting (DOWN) paths for each gene group (oncogenes in pink, onco-suppressors in blue), and each pathway was plotted as bar graphs enclosed in rectangular frames. The bars in orange represent the average of an equivalent fraction in 1000 different random collections of 700 genes. Significance of the observed differences was evaluated by a two-sided t-test (*p* value: * <0.025, ** <0.0025, *** <0.00025, **** <0.000025).

As the paths that link genes to rate limiting enzymes are “signed” we could also ask whether onco-genes or TSG are more likely to upregulate or downregulate each of the targeted metabolic pathways. To this end we considered the paths that link the lists of oncogenes and onco-suppressors to metabolic pathways and we classified them as activating or inhibiting depending on whether they contained an even or odd number of inhibitory steps. Next, we asked which pathways are more likely to be up or down regulated by oncogenes and TSG (Figure 2). Consistently with the notion that oncogene expression tends to stimulate aerobic glycogenesis at the expense of oxidative phosphorylation (Yu et al., 2017), by this analysis 75% and 70% of paths predicted to positively regulate glycolysis and negatively regulate the citric acid cycle start from an oncogene. Conversely, TSG are predicted to have an opposite impact on glucose utilization (Figure 2). In addition, cancer genes are also predicted to activate various metabolic pathways, including those generating nucleosides and amino acids to facilitate the biosynthesis of macromolecules and organelles required for building new cells.

Our graph approach not only suggests whether a cancer gene has the potential to modulate a metabolic pathway but also precisely points to the molecular steps underlying such inferred modulation and lists the drugs or other chemicals targeting the activity of the proteins in the path. More than 8,600 causal paths connecting cancer genes to rate limiting metabolic genes are predicted by the graph algorithm that we have developed. As an example, the Fms-like tyrosine kinase 3 gene (FLT3) that, when activated by the ITD oncogenic mutation, promotes aerobic glycolysis is predicted by our algorithm to do so via AKT-mediated upregulation of hexokinase. This prediction is consistent with previously reported experimental findings (Ju et al., 2017). A detailed discussion of this information is beyond the scope of this report but interested readers can freely inspect this information rich dataset by interrogating the CancerGeneNet resource.

## Conclusion

Metabolic changes in tumours have long been acknowledged (Warburg 1956; Nanda et al., 2020) and metabolic pathways are considered promising therapeutic targets (Batra et al., 2013). However, the mechanistic details of the crosstalk between the activity of the signalling pathways that are affected by oncogenes or onco-suppressors and metabolism have not been clearly defined. This limits our ability to design targeted therapeutic interventions.

An in-depth understanding of the functional interactions underlying cancer metabolic reprogramming is essential to identify cellular vulnerabilities that can be exploited for therapeutic opportunities (Wolpaw and Dang 2018). Here we have combined a curation effort to annotate experimental evidence linking cell signalling and metabolism with the development of graph algorithms, thus providing a tool that supports the inference of causal paths underlying the molecular mechanisms that explain the impact of gene mutations on cell metabolism.

The results of this approach are made available via interrogation of the CanceGeneNet resource (https://signor.uniroma2.it/CancerGeneNet) where it is possible to visualize the paths connecting genes to activity of rate limiting enzymes. The resource can be interrogated with a query protein or a list of proteins and results are displayed either graphically or in a tabular format.

The curation work described in this report represents an important addition to the functional network represented in the SIGNOR resource that goes beyond mere consultation. The integrated signaling-metabolic network supports users in the interpretation of experimental results (e.g., proteomics, phosphoproteomics or metabolomics) by offering a framework that combines signaling and metabolism. The resource has been primarily conceived to model how the signal transduction cascades, triggered by oncogenes and TSG, modulate the metabolic profile of malignant cells. However, it is not limited to this scope, as the interactome that is available for download in the CancerGeneNet resource contains causal paths linking any gene in SIGNOR to metabolic pathways, thus supporting the integration of omic data in a variety of biological contexts. To this end, it would be of interest to expand the landscape of metabolic pathways integrated in the resource.

When examining the paths by the approach described here, users should consider that the SIGNOR human interactome is assembled from observations in a variety of experimental systems. Not all molecular connections that are annotated in the resource are active in all cell contexts because either one of the proteins is not present or not active in the specific cell type. Thus, each proposed molecular path should be critically examined by domain experts. In principle gene expression evidence could be exploited to filter out paths that pass over nodes representing proteins that are not expressed in the biological system of interest. Nevertheless, given that a number of possible paths are suggested by interrogating the resource, domain-experts are in the position to identify paths that are worth testing experimentally.

## Methods

### Input Datasets

The cancer-driver gene list (v95) was downloaded from the Cancer Gene Census (CGC) web site (Sondka et al., 2018). The CGC associates 729 genes to 365 tumour types. 490 of these genes are unambiguously classified as oncogenes (245) or TSG (tumour suppressor genes) (245).

The strategy to find functional paths connecting cancer genes to metabolic enzymes builds on a human causal network assembled from the causal interactions captured from the literature and annotated in the SIGNOR resource (Licata et al., 2020). SIGNOR curators capture both direct (i.e. MEK phosphorylates and activates ERK) and indirect interactions (i.e. EGF stimulation enhances the activation of ERK). The human interactome assembled for the analysis reported here only includes direct interactions annotated in the February 2022 version of SIGNOR. This dataset can be downloaded from the SIGNOR website and is represented as a signed directed graph of 8,486 nodes and 30,930 edges. Each causal relationship in SIGNOR 2.0 is associated with a score reflecting an estimate of its functional relevance and experimental support.

### SIGNOR Score

Interactions in SIGNOR are assigned a significance score, ranging from 0.1 to 1. Interactions between proteins, protein families and complexes are assigned a significance score calculated by using a principal component regression (PCR) approach (Mevik and Wehrens 2007) which yields a predictive model considering, as supporting features, the number of experimental evidence in SIGN.

OR and score values extracted from the STRING database (Szklarczyk et al., 2021). The model was refined by optimizing its ability to predict the interactions participating in a molecular pathway annotated in our resource. Interactions involving the remaining classes of SIGNOR entities, namely small molecules, phenotypes, stimuli, chemicals, fusion proteins, miRNAs and antibodies are assigned an arbitrary score (e.g., interactions involving chemicals and small molecules are assigned a default significance score of 0.8). Additional details are available at https://signor.uniroma2.it/documentation/.

### Estimating the Impact of a Protein on a Metabolic Pathway

To investigate the regulatory impact of a protein (or a list of proteins) on a metabolic pathway, we make use of the graph representation of the human causal network annotated in SIGNOR 2.0. We developed a tool that, for each query node and metabolic pathway, “navigates” the network graph and retrieves the shortest paths connecting the query node to a rate-limiting enzyme of the end pathway. Shortest paths are identified using a strategy adapted from Perfetto et al. (2021). For each query protein and metabolic pathway, we retrieve all the directional paths (of length of four steps or fewer) connecting the query protein and the rate-limiting enzymes (RLEs) of the pathway. As any step in the graph has a score (s), we define the distance between any two interacting proteins as d = 1- s and the path distance score of a path including more than two nodes as the sum of the distance of the edges forming the path. The lower the path distance score, the shorter is the “functional distance” and more functionally relevant is estimated the path. Next, we classify as proteins having an influence on a metabolic pathway activity those proteins having Z-score in the path distance distribution lower than Z = −1.96 (*p*-value <0.05) The Z-score is computed over the distribution of path distance scores considering the distance between every protein in SIGNOR to the RLEs of any given pathway. Nodes that cannot connect with four steps or fewer to RLEs were arbitrarily assigned the highest path distance score in the distribution.

The paths connecting the query protein to a metabolic pathway are characterized by a distance and by a sign that specifies whether the protein is inferred to have a positive or a negative effect on pathway activity. Proteins connected by paths formed by an odd number of inhibitory steps are defined as inhibitors, otherwise are considered as activators.

Identification of the paths linking a query gene to any gene annotated to a pathway was programmatically implemented using the “all_simple_paths” function of the NetworkX module of the Python language (Hagberg et al., 2008). The function returns every short path linking any two nodes in an oriented graph. We set a length cut-off (cut-off 4) as input parameter in order to explore only pathways with a length that is shorter or equal to the chosen threshold. R scripting was used to run python scripts and to analyse results. The tool is made available in the CancerGeneNet resource (https://signor.uniroma2.it/CancerGeneNet/) (Iannuccelli et al., 2020).

### Metabolic Impact of Oncogenes and TSG

To identify the impact of oncogenes and tumour suppressor genes (TSG) on the modulation of metabolic pathways we used the strategy outlined in the previous section to link to the up- or down-regulation of 10 metabolic pathways, 245 oncogenes or 245 TSGs as annotated in the Cancer Gene Census (CGC) resource. We used the two-sided t-test to assess whether the proportion of paths starting from oncogenes/TSGs and leading to the up-/down-regulation of a key metabolic pathway is significantly greater or smaller than the mean extracted from a randomized dataset. Briefly, we generated lists of 700 genes that were randomly chosen among the genes annotated in SIGNOR. 700 corresponds to the number of genes in the CGC resource. We generated 1000 of such random genes lists and, for each, we evaluated the fraction of short paths (<= 4 steps) starting from the input genes and impacting the up- or down-regulation of the key metabolic pathways. We eventually charted the distribution of these fractions and performed a t-test.

To facilitate the interpretation of the results, the SIGNOR pathway “glycolysis and gluconeogenesis” was demerged into two sub-pathways: “Glycolysis” and “Gluconeogenesis.”

## Data Availability

The datasets presented in this study can be found in online repositories. The names of the repository/repositories and accession number(s) can be found below: The dataset generated for this study can be found in the CancerGeneNet resource, https://signor.uniroma2.it/CancerGeneNet/, Download section.
